# Retraction: Knockdown of long non-coding RNA KCNQ1OT1 restrained glioma cells' malignancy by activating miR-370/CCNE2 axis

**DOI:** 10.3389/fncel.2023.1181681

**Published:** 2023-03-24

**Authors:** Frontiers Editorial Office

Following publication, concerns were raised regarding the integrity of the images in the published figures. The authors failed to provide a satisfactory explanation during the investigation, which was conducted in accordance with Frontiers' policies.

The retraction was approved by the Chief Editors of Frontiers in Cellular Neuroscience and the Chief Executive of Frontiers. The authors did not agree to the retraction.

